# Supplementary material for: Tip dating supports novel resolutions of controversial relationships among early mammals
Source: Proc Biol Sci. 2020 Jun 10;287(1928):20200943. doi: 10.1098/rspb.2020.0943 (PMC7341916; doi:10.1098/rspb.2020.0943)
Supplement: Supplementary text, figures, methods and references [file rspb20200943supp1.pdf]

Supplementary information

Tip dating supports novel resolutions of controversial relationships among early mammals

Benedict King and Robin M. D. Beck

Contents

**Page 2**	Effects of stratigraphic age and branch length on tree topology
**Pages 3–4**	Supplementary Figures 1–2
**Page 5**	Relationship of *Haramiyavia*, *Thomasia* and tritylodontods
**Page 5**	Effect of fossil sampling on age estimates of *Juramaia* and *Rugosodon*
**Pages 6–15**	Supplementary figures 3–13
**Page 16**	Further details on priors used for tip-dated analysis
**Pages 17–30**	Sources for taxon ages
**Pages 31–37**	Supplementary references

Effects of fossil age and branch length on tree topology

To test the effect of taxon age on the phylogenetic position of haramiyidans, we ran an analysis without data on a partially fixed topology (based on the result from tip dating) in which only the haramiyidan taxa (i.e. *Haramiyavia*, *Thomasia* and the euharamiyidans) were free to move around the tree. Specifically, a series of backbone constraints based on the maximum clade credibility tree from the main analysis were implemented, and haramiyidans were constrained to form two monophyletic groups (the Triassic *Haramiyavia* + *Thomasia,* and the Middle Jurassic euharamiyidans) but with their phylogenetic position otherwise unconstrained. The analysis therefore tested where these two groups attached to the backbone, based purely on their stratigraphic ranges. Processing of results in R required the packages ape (Paradis et al. 2004), phangorn (Schliep 2010) and treeio (Yu et al. 2017).

Placement of the Middle Jurassic euharamiyidans (figure S1a) based purely on stratigraphic data (referred to hereon as the stratigraphic phylogenetic position) is very different from that of the Late Triassic *Haramiyavia* and *Thomasia* (figure S1b). The stratigraphic phylogenetic position for *Haramiyavia* and *Thomasia* is concentrated around the very oldest part of the tree: the time at which *Haramiyavia* and *Thomasia* branch from the rest of the tree has a 95% highest posterior density (HPD) interval of 203.5 – 237.1 Ma. The stratigraphic phylogenetic position for euharamiyidans is concentrated on younger branches (HPD 171.4 – 215.2 Ma). Notably, both groups are also more likely to attach to longer branches (figure S1). When the stratigraphic phylogenetic position is corrected for the effect of branch length, by dividing probability by branch length (figure S2), the temporal signal is more obvious. Quantification of the probability that each clade occurs above a particular node also shows that the stratigraphic phylogenetic position for *Haramiyavia* and *Thomasia* is strongly concentrated at the very base of the tree, in contrast to the euharamiyidans (figure S1c).

These results show that there are two major factors (beyond morphology) in determining phylogenetic position in tip-dated analyses: age and branch length. Long branches tended to 'attract' the clades under investigation, but only long branches that occurred in an appropriate age range. This supports the suggestion of Turner et al. (2017) that Bayesian tip-dated approaches disfavour long un-sampled branches.

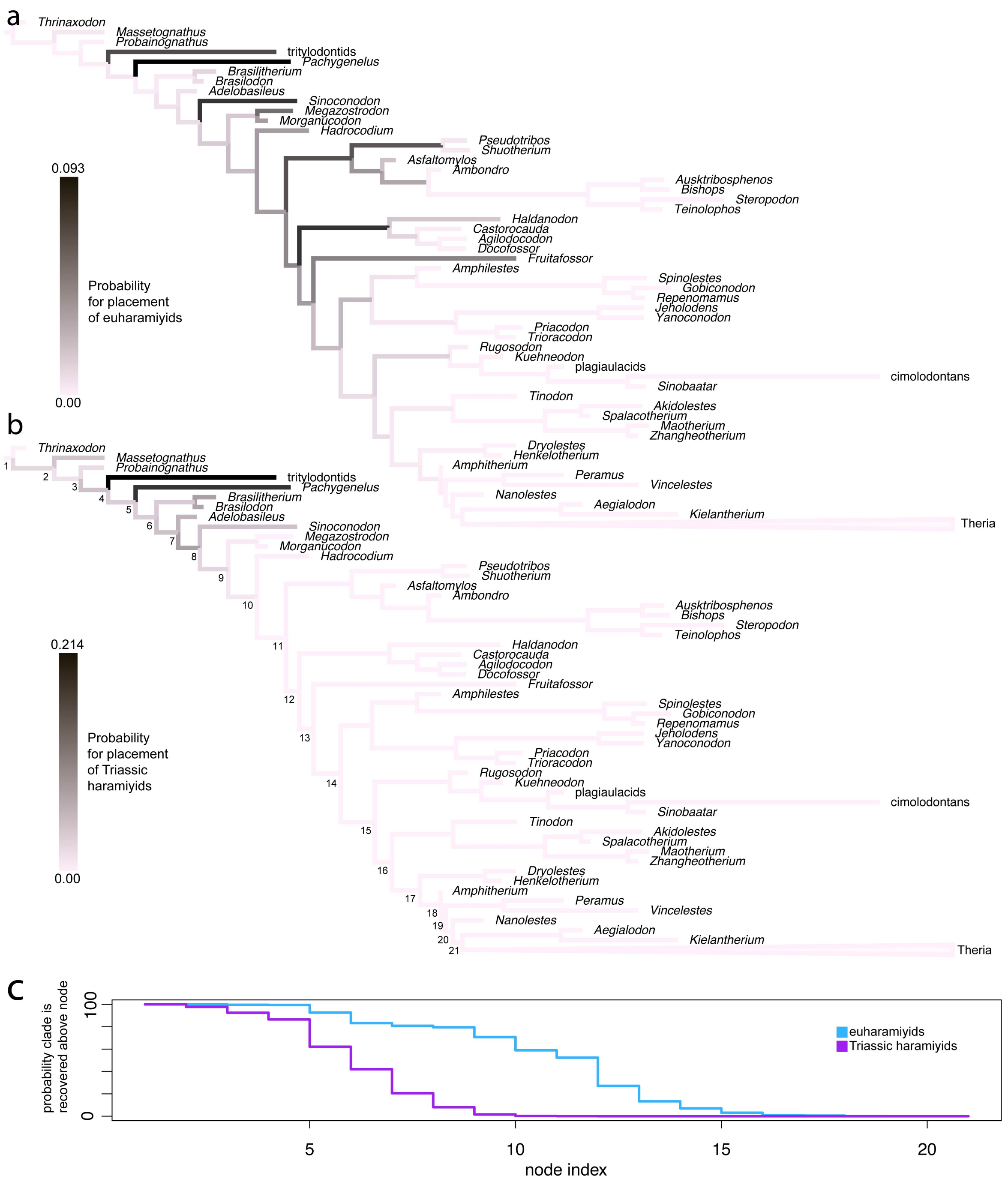

Supplementary Figure 1. Phylogenetic position of haramiyidans based only on stratigraphic ranges. A-B) The tree is a fixed backbone constraint based on the maximum clade credibility tree from the main analysis, on which the branching positions for the two groups of haramiyidans are estimated. Branch colours represent the probability that the respective clade (A, Euharamiyida; B, *Haramiyavia*+*Thomasia*) was found on each branch in an analysis run without morphological data. The probabilities for *Haramiyavia* and *Thomasia* are concentrated at the base of the tree, unlike the probabilities for euharamiyidans which are more diffuse and centred in younger parts of the tree. There is also a notable preference for long branches. C) Probabilities that each group of haramiyidans is found above each node in a sequence from the base of the tree to the crown therian node. The distribution for *Haramiyavia*+*Thomasia* drops off much more quickly, representing the expectation based on their age that they are placed in a lower position on the tree than euharamyidans.

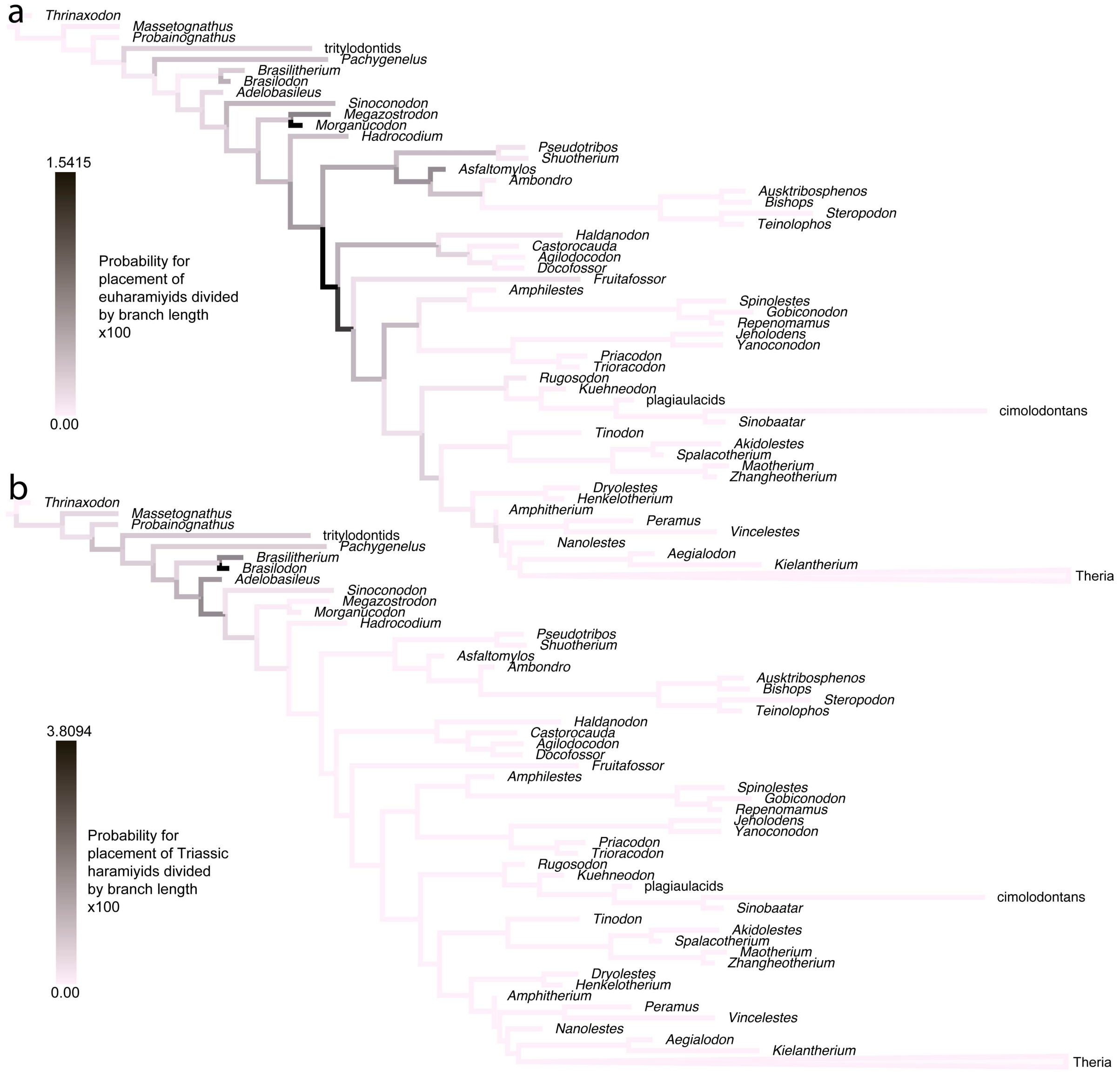

Supplementary Figure 2. Effective topology prior for haramiyids, corrected for branch length. a-b) The tree is a fixed topology based on the maximum clade credibility tree from the main analysis, on which the prior probabilities for the position of two groups, divided by the branch length, of haramiyids are mapped. Branch colours represent the corrected prior probability that the respective clade (a, euharamiyids; b, Triassic haramiyids) was found on that branch in an analysis run without data. The corrected prior probabilities for Triassic haramyids are concentrated at the base of the tree, unlike the corrected prior for euharamiyids, which is more diffuse and centred in younger parts of the tree. Removing the effect of branch lengths clearly shows the temporal signal in the prior.

Relationship of *Haramiyavia, Thomasia* and tritylodontods

A surprising aspect of our results is the placement of *Haramiyavia* and *Thomasia* with tritylodontids (fig. 2). This has been found in some previous analyses (e.g. Gurovich and Beck 2009), but it should be noted that these two groups show marked dental differences (Kemp 1982; Jenkins et al. 1997; Kielan-Jaworowska et al. 2004; Luo et al. 2004; Kemp 2005; Luo et al. 2015a; Velazco et al. 2017). The upper postcanines of tritylodontids comprise three major rows of cusps arranged labiolingually, whereas those of *Haramiyavia* and *Thomasia* have only two. Tritylodontids show a specialised type of postcanine dental replacement, in which worn teeth shed from the anterior end of the tooth row and new teeth are added at the posterior end, whereas this is absent in *Haramiyavia* (the pattern of dental replacement in *Thomasia* is unknown). *Haramiyavia* also retains the upper and lower canines, whilst these teeth have been lost by tritylodontids. Based on these striking morphological differences (only some of which were used as characters by Huttenlocker et al. (2018)), this relationship should be viewed cautiously, pending description of additional cranial and postcranial material of *Haramiyavia*, and *Thomasia*, and improved taxon and character sampling of non-mammaliaform cynodonts. As discussed above, the recovery of this relationship is likely driven by the relatively long branch leading to tritylodontids, which also means that morphological differences between tritylodontids and *Haramiyavia* and *Thomasia* are penalised less.

Effect of fossil sampling on age estimates of *Juramaia* and *Rugosodon*

The result of the age estimate of *Juramaia* is striking (Fig. 3). However, it is possible that poor sampling of eutherians in the Early Cretaceous drives this result. In order to test this idea, we manufactured an equivalent situation for the multituberculate *Rugosodon* by deleting *Kuehneodon* and plagiaulacids from the dataset. This produces an approximately 40 million-year gap between *Rugosodon* from the Yanliao biota and *Sinobaatar* from the Jehol biota, equivalent to the temporal difference between *Juramaia* and *Eomaia*. We then estimated the age of *Rugosodon* using the same laplace distribution prior as used for other taxa from the Yanliao biota (main text: Material and Methods).

Estimating the age of *Rugosodon* following deletion of *Kuehneodon* and plagiaulacids resulted a younger age estimate (Fig. S12), suggesting that sampling issues may indeed affect the age estimate for *Juramaia.* However, the results are not entirely equivalent. For *Rugosodon*, the upper bound of the HPD (114.4–164.5) still overlaps with the correct age, whereas the upper bound of the HPD for *Juramaia* is much younger (106.3–137.6). Deletion of taxa results in a loss of precision for the age estimate of *Rugosodon*, representing a lack of information in the data. In contrast, the age estimation for *Juramaia* results in a distinct Early Cretaceous peak (Fig. 3), showing a strong signal in the morphological data. Nevertheless, these results show the importance of fossil sampling to constrain estimates of taxon age, and suggest the implications of the morphology of *Juramaia* may be subject to change if additional Early Cretaceous eutherian fossils are discovered.

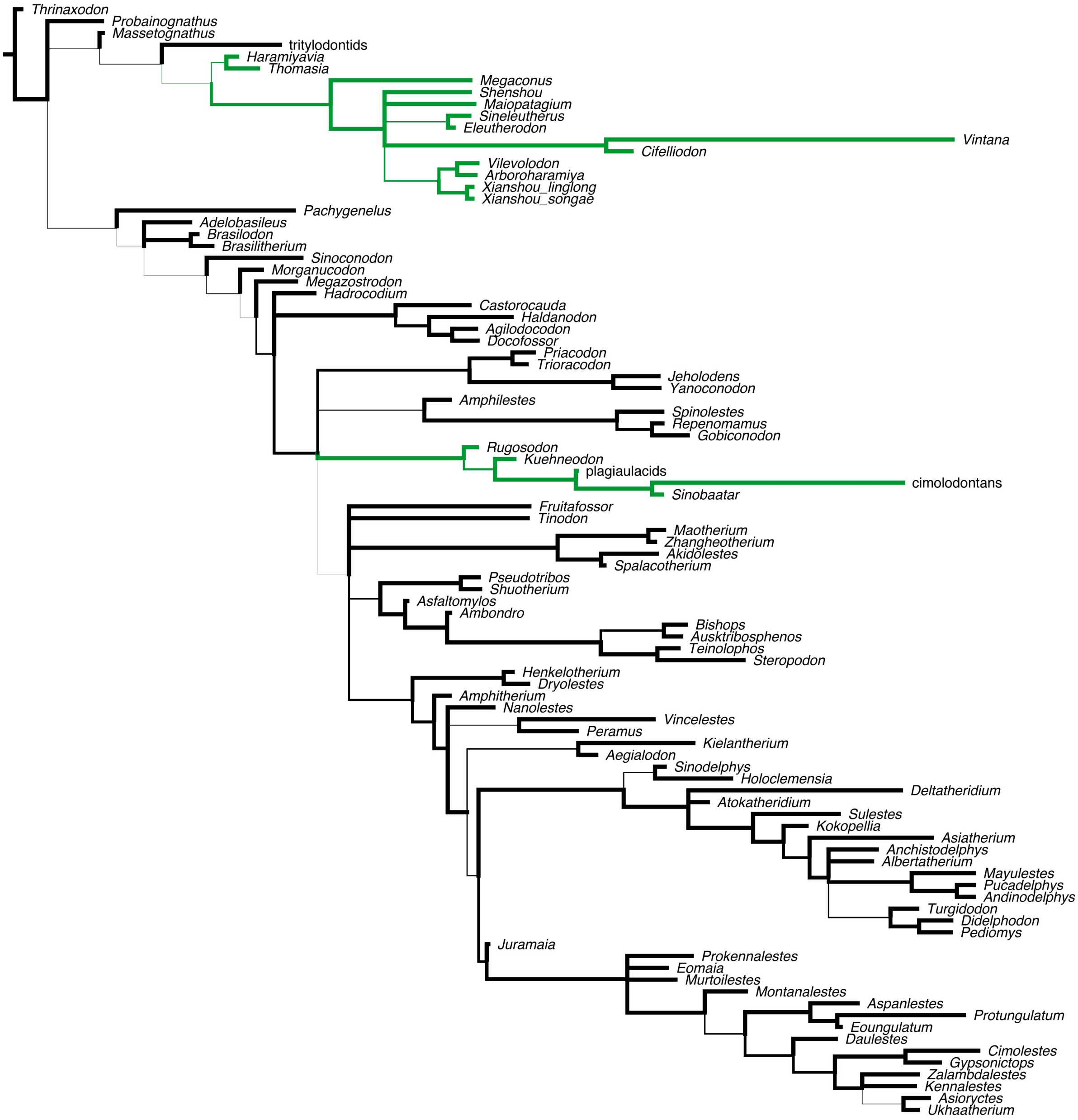

Supplementary Figure 3. 50% majority rule consensus tree using craniodental data only. Tip-dated analysis from BEAST2 on the dataset of Huttenlocker *et al.* (2018). 'Allotherian' taxa in green. In contrast to the full dataset, this analysis has increased support for haramiyidan monophyly.

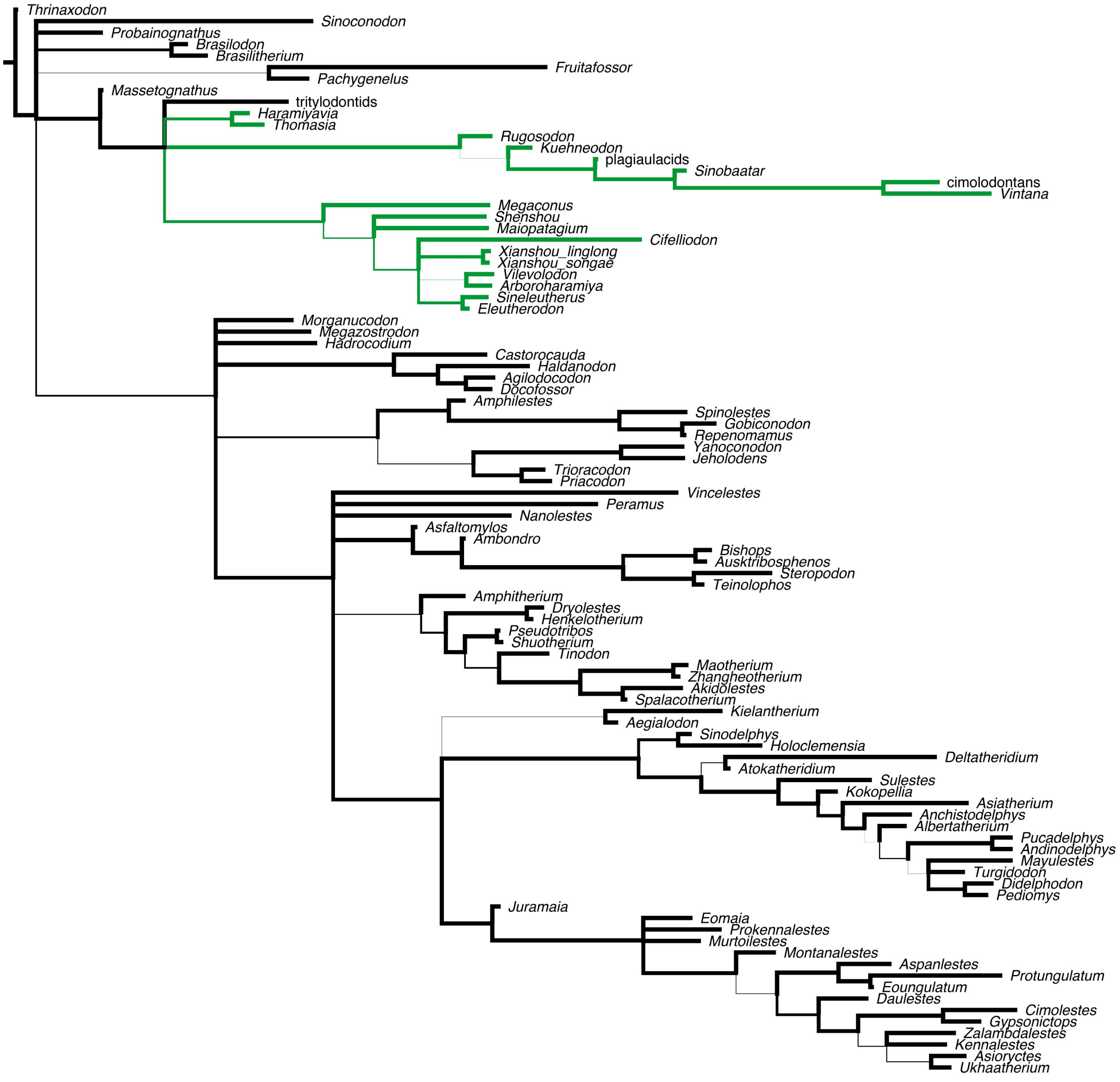

Supplementary Figure 4. 50% majority rule consensus tree using dental data only. Tip-dated analysis from BEAST2 on the dataset of Huttenlocker *et al.* (2018). ‘Allotherian’ taxa in green. This analysis shows support for allotherian monophyly.

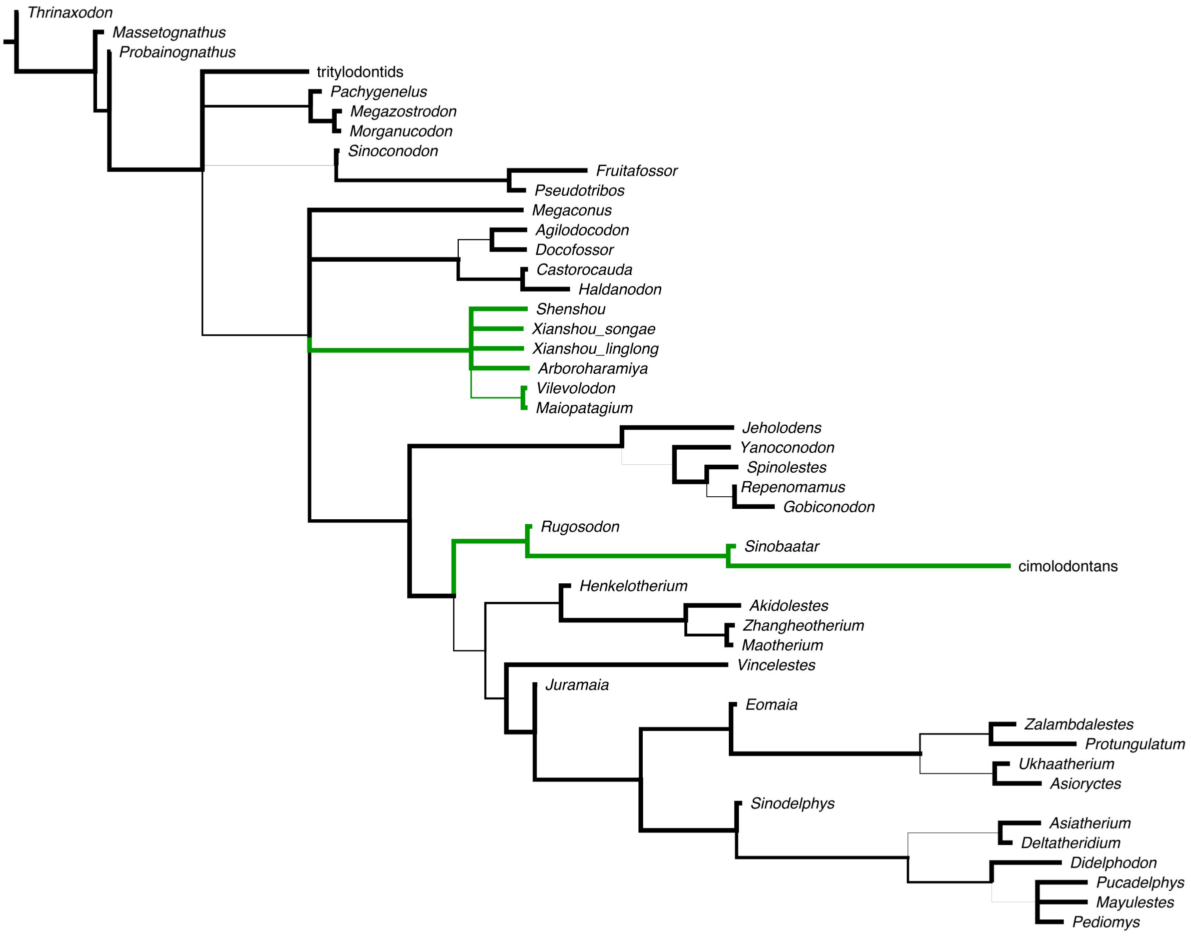

Supplementary Figure 5. 50% majority rule consensus tree using postcranial data only. Tip-dated analysis from BEAST2 on the dataset of Huttenlocker *et al.* (2018). 'Allotherian' taxa in green. This analysis separates euharamiyids from multituberculates, but cannot be used to investigate the position of Triassic haramiyidans.

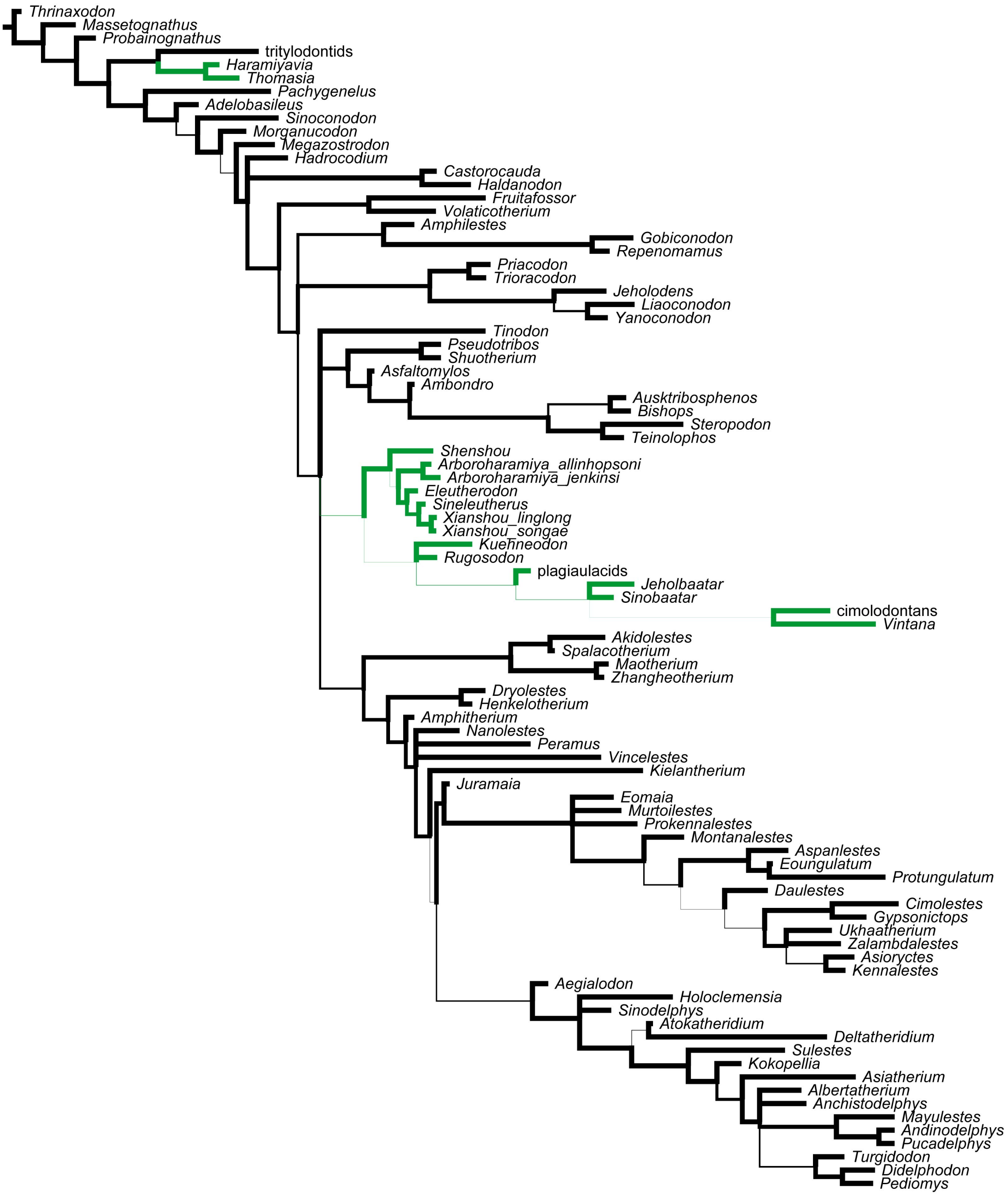

Supplementary Figure 6. 50% majority rule consensus tree using the dataset of Wang *et al* (2019). Tip-dated analysis from BEAST2. 'Allotherian' taxa in green. This analysis separates euharamiyids from *Haramiyavia* and *Thomasia*, while euharamiyids and multituberculates form a clade.

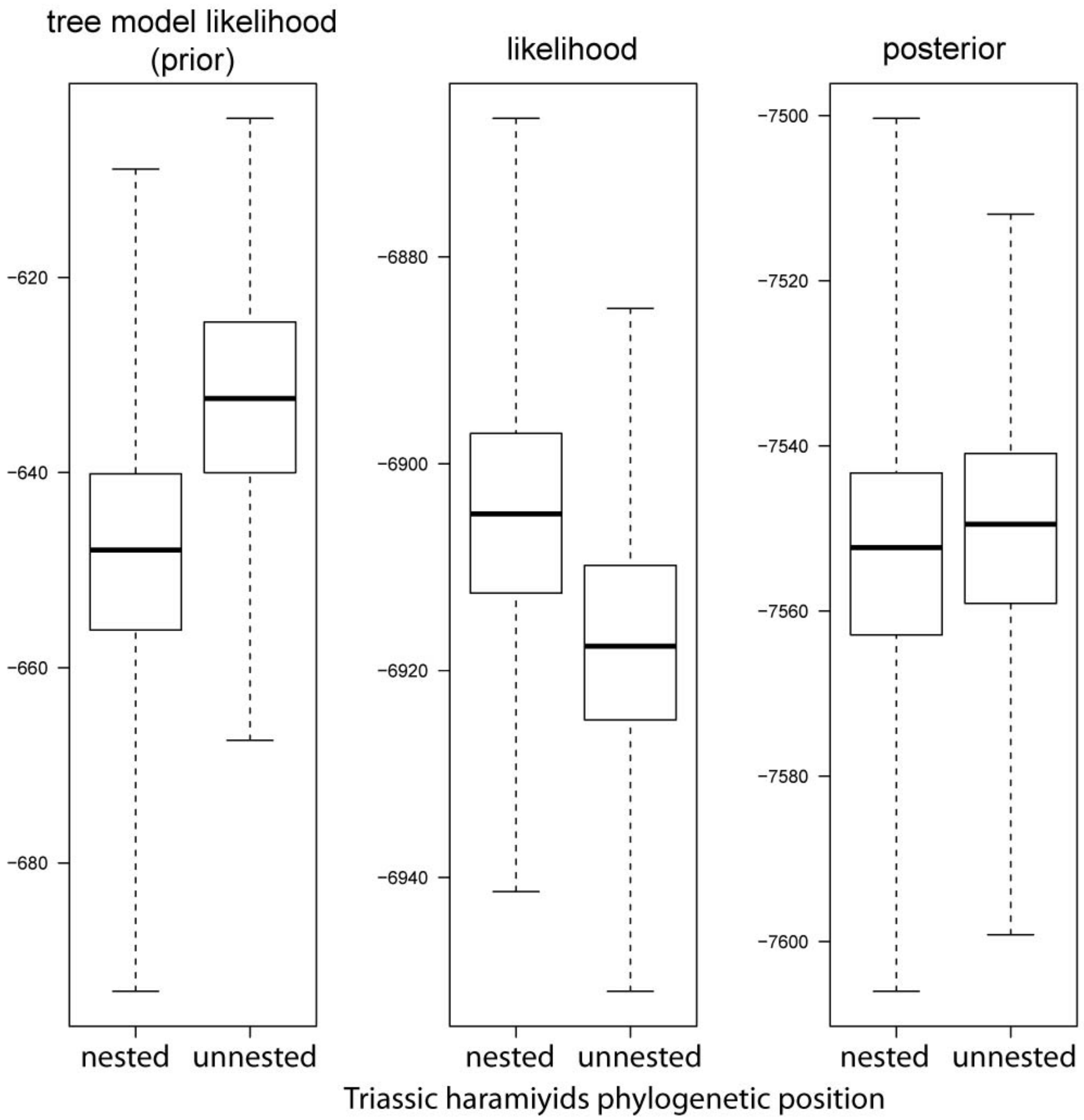

Supplementary Figure 7. Twin likelihood peaks in the tip-dated analysis on the Krause *et al.* (2014) dataset. Boxplots represent the calculated prior (tree model likelihood), likelihood and posterior probabilities within the two likelihood peaks, which correspond to alternative phylogenetic positions for the Triassic haramiyids. An un-nested position has a higher prior probability (due to improved stratigraphic fit of the resulting phylogenies), but a lower likelihood (due to lower congruence with the morphological data, particularly the dental data). The contradictory effects of the prior an likelihood on the two topologies lead to approximately equal posterior probabilities, and both were therefore sampled during the analysis.

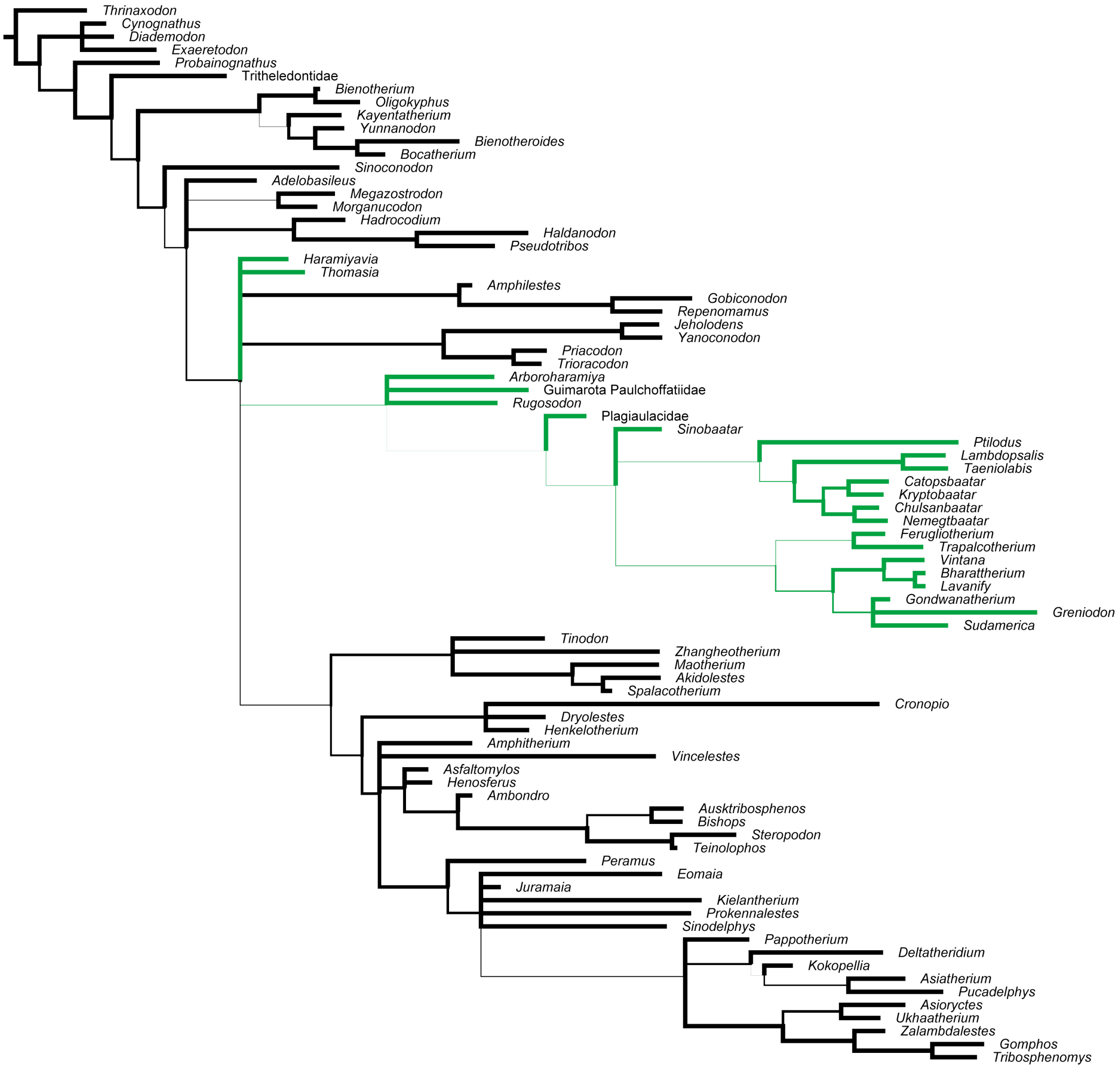

Supplementary Figure 8. 50% majority rule consensus tree for the Krause *et al.* (2014) dataset. Tip-dated analysis from BEAST2. 'Allotherian' taxa in green.

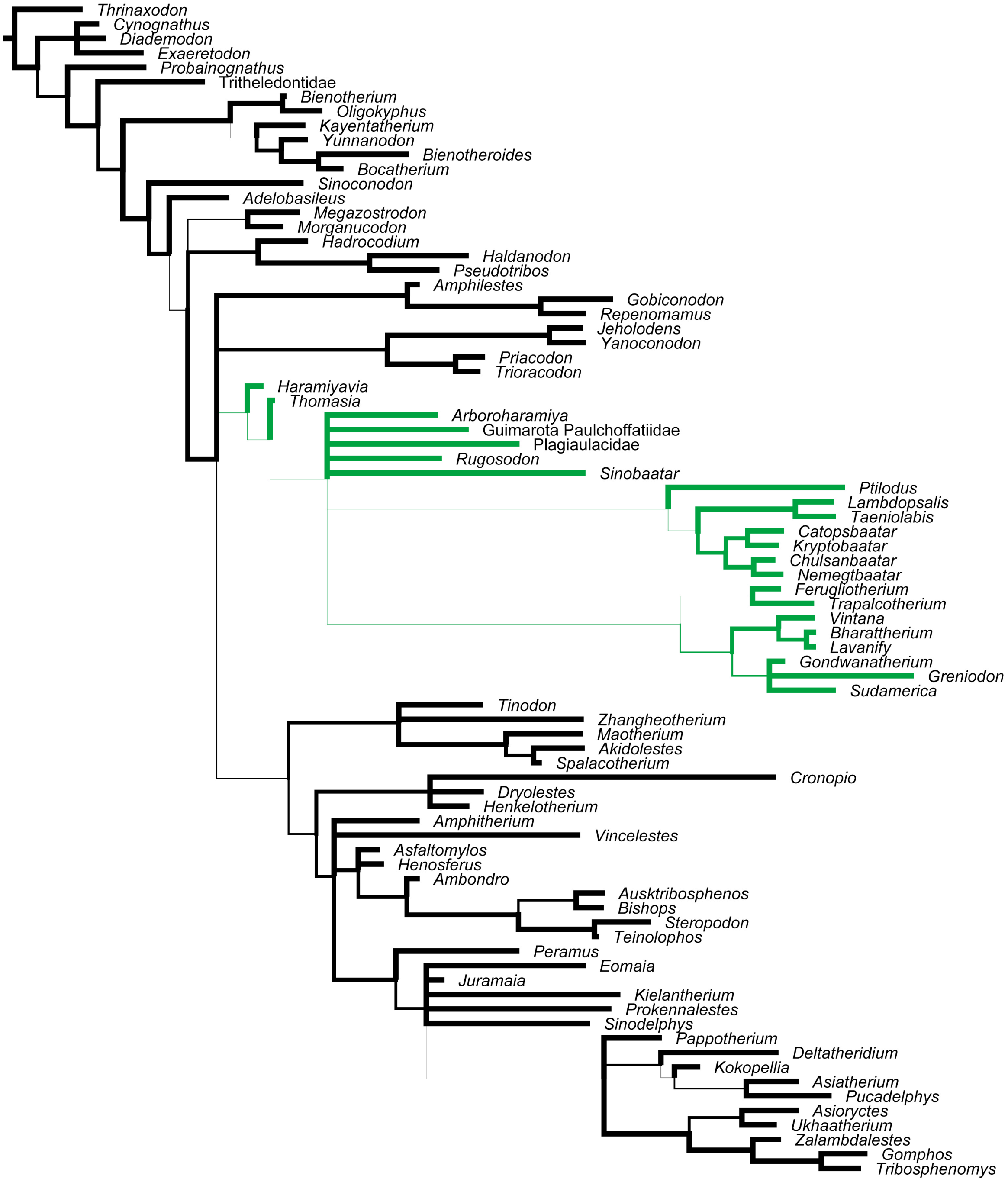

Supplementary Figure 9. 50% majority rule consensus tree for the Krause *et al.* (2014) dataset, using only the trees from the posterior sample that recovered Triassic haramiyids in a nested position. Tip-dated analysis from BEAST2. ‘Allotherian’ taxa in green.

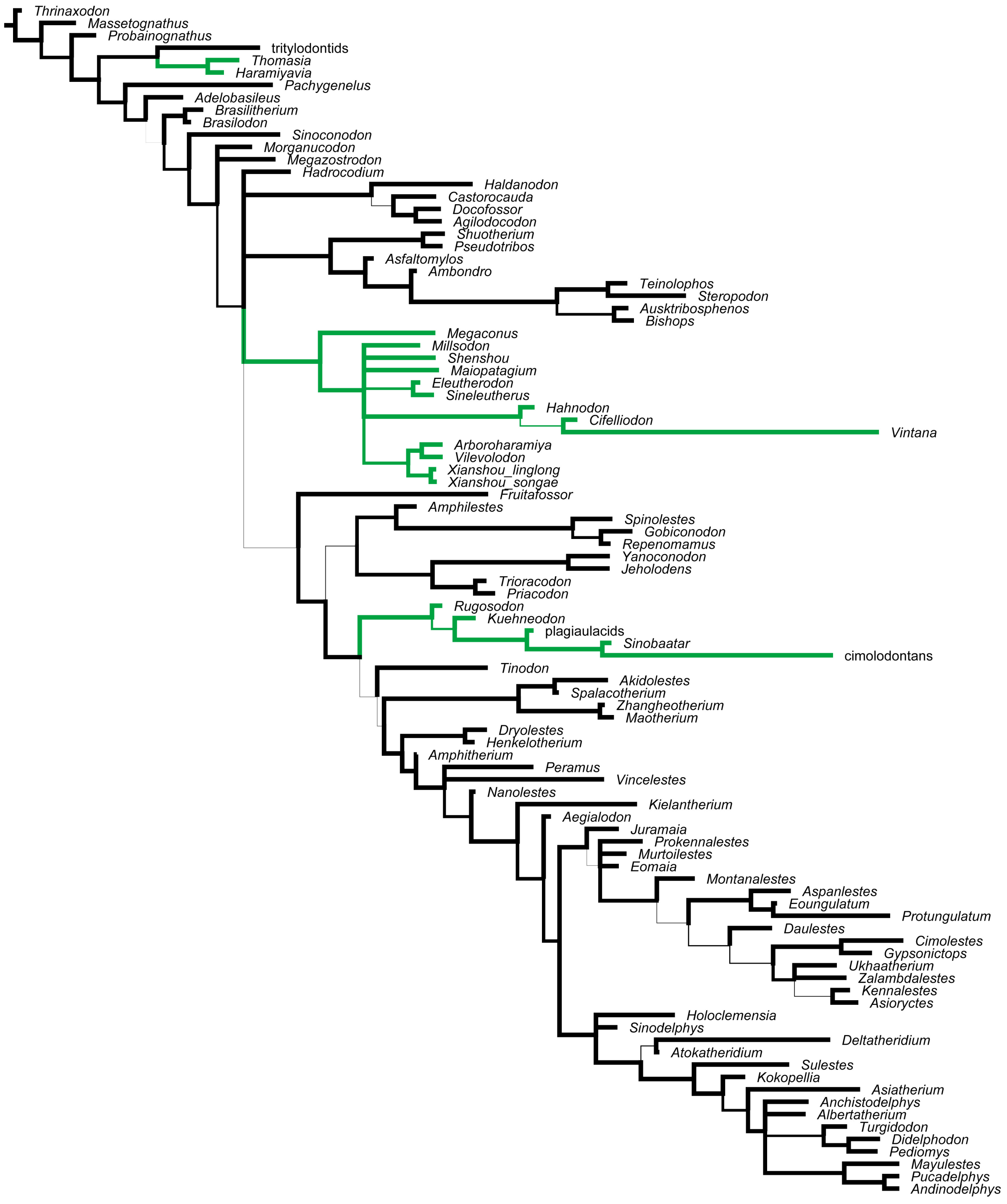

Supplementary Figure 10. 50% majority rule consensus tree for the Krause *et al.* (2014) dataset, using only the trees from the posterior sample that recovered Triassic haramiyids in an un-nested position. Tip-dated analysis from BEAST2. ‘Allotherian’ taxa in green.

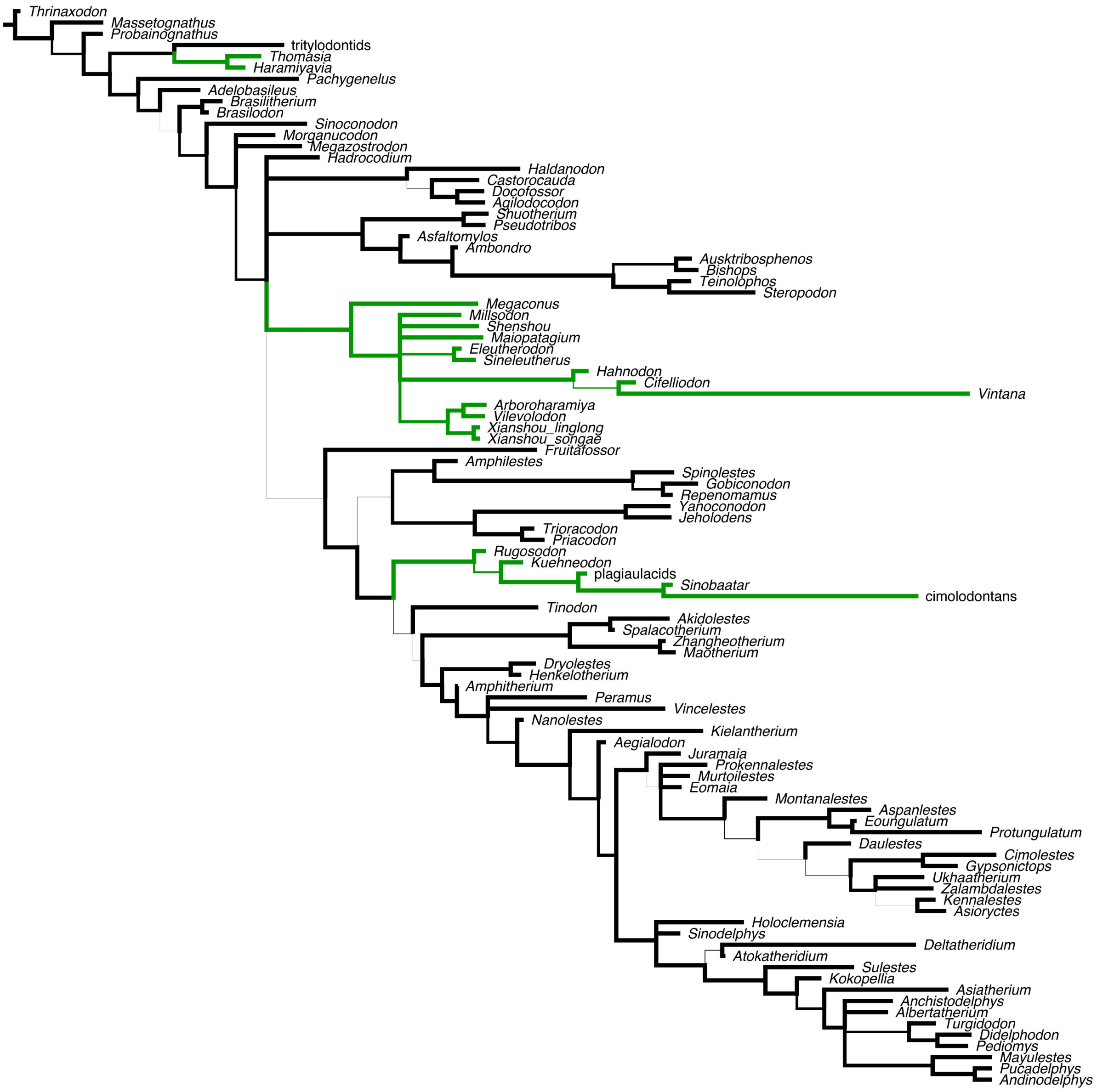

Supplementary Figure 11. 50% majority rule consensus tree for the Huttenlocker *et al.* (2018) dataset, where the age of *Juramaia* was allowed to vary. Tip-dated analysis from BEAST2. 'Allotherian' taxa in green.

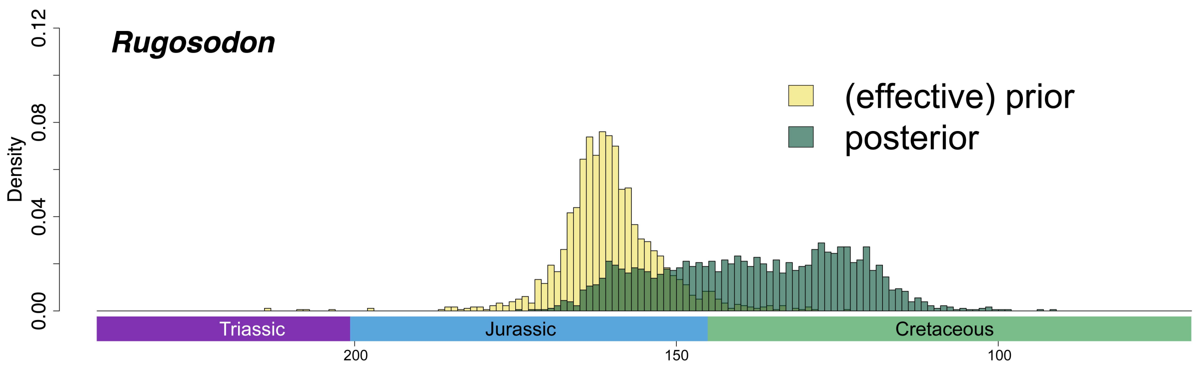

Supplementary Figure 12. Age estimate for *Rugosodon* after removal of *Kuehneodon* and plagiaulacids. This leads to a younger and much less precise age estimate, and suggests that the young age estimate for *Juramaia* could be partly driven by the lack of sampling of eutherians in the Early Cretaceous.

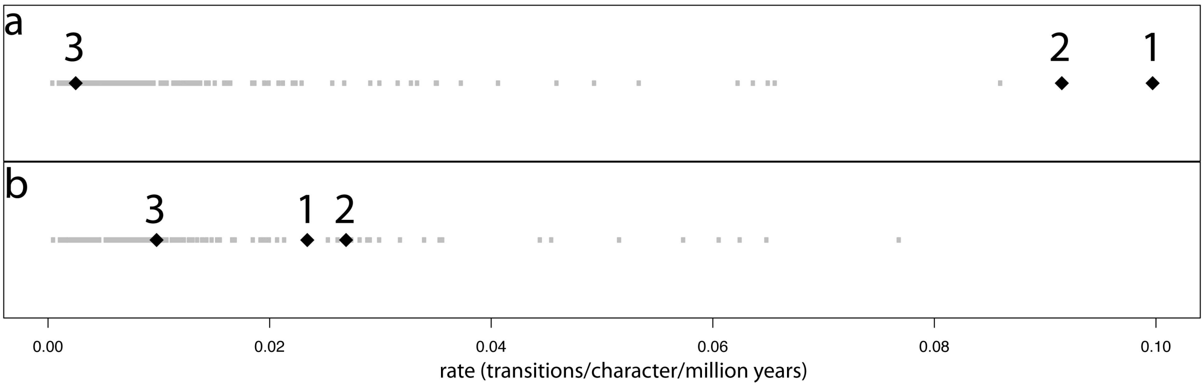

Supplementary Figure 13. The Jurassic age of *Juramaia* necessitates highly heterogenous rates of evolution. a) Rates of evolution on each branch from the majority rule consensus tree of the main analysis (*Juramaia* assigned a Jurassic age). Rates from three key branches are highlighted: 1, the branch leading to the therian crown node; 2, the branch leading to the common ancestor of eutherians; and 3, the branch leading to eutherians excluding *Juramaia*. There is a large reduction in the rate of evolution following the divergence of *Juramaia* from the rest of the eutherians. b) Rates of evolution on each branch from the majority rule consensus tree of the analysis in which the age of *Juramaia* was allowed to vary (and was estimated to be Early Cretaceous). The rates on the same three branches are much more similar.

Further details on priors used for tip-dated analysis

The analyses did not use a diversified sampling model (Zhang et al. 2016), as this is only relevant when extant taxa are sampled. The datasets were modified so that extant taxa and recent fossils were removed.

Details of parameters and priors. Lognormal distributions are in log space.

**Parameter**	**Value**
Removal probability	Fixed (0.0)
Origin time	Uniform (0, 2000)
Birth Rate	Lognormal (mean -2, sd 1)
Death Rate	Exponential (mean 0.1)
Sampling Rate	Exponential (mean 0.1)
Gamma shape	Uniform (0, 10)
Clock Rate	Lognormal (mean -5, sd 1.4)
Clock standard deviation	Exponential (mean 1)

Tip ages

Here we give the ages of each taxon, with references. These were assigned as uniform priors in the analysis. Occurrences are taken from the literature, or from the Paleobiology Database (accessed through Fossilworks http://www.fossilworks.org). Occurrences using the Paleobiology database are referenced with "PBDB". Occurrences from the literature and the PBDB are recorded according to Geological stage, and for standardisation were converted to age using the International Chronostratigraphy Chart (Cohen et al. 2018). Occurrences on the PBDB listed as *cf. Taxon* were not included. Regional stages were converted to international stages based on the Geowhen database (http://www.stratigraphy.org/bak/geowhen/index.html). When sources referenced subdivisions of stages (e.g. early, middle, late Campanian), the stage was divided into three equal-sized intervals. Radiometric dates are used, when available.

Taxa from Huttenlocker *et al.* dataset

Adelobasileus
Tecovas formation. Norian.
(Lucas and Luo 1993; Parker et al. 2008)

Aegialodon
Cliff end, Wadhurst formation. Early Valanginian.
(Kielan-Jaworowska et al. 2004)

Agilodocodon
Daohugou locality, Tiaojishan formation
(Meng et al. 2015)

Akidolestes
Yixian formation. Radiometric date 129.7 (base), and 122.1 (top)
(Li and Luo 2006; Chang et al. 2009)

Albertatherium
Deadhorse Coulee Member (Milk River Formation). Late Santonian
Deadhorse Coulee Member (Eagle Formation). Santonian
PBDB

Ambondro
Isalo group. Bathonian.
(Flynn et al. 1999)

Amphilestes
Taynton Limestone formation (Stonesfield slate), Middle Bathonian.
(Butler and Sigogneau-Russell 2016)

Amphitherium
Taynton limestone formation (stonesfield slate), Middle Bathonian.
(Butler and Clemens 2001)

Anchistodelphys
Smoky Hollow Member (Straight Cliffs Formation). Late Turonian
Wahweap Formation, Early/Lower Campanian

Andinodelphys
Tiupampan mammal zone, Santa Lucía Formation, Danian
PBDB

Arboroharamiya
Yanliao biota, 166-159
(Zhou and Wang 2017)

Asfaltomylos patagonicus
Canadon asfalto formation, Toarcian.
(Rauhut et al. 2002; Cúneo and Bowring 2010)

Asiatherium
Barun Goyot formation equivalent. Campanian-Maastrichian.
(Szalay and Trofimov 1996; Longrich et al. 2010)

Asioryctes
Barun Goyot formation. Campanian-Maastrichian.
(Kielan-Jaworowska 1975)

Aspanlestes
Darbasa Formation, Early/Lower Campanian
Aitym Formation, Late/Upper Turonian to Late/Upper Turonian
Bissekty Formation, Coniacian
PBDB

Atokatheridium
Middle Member (Antlers Formation), Late/Upper Aptian to Late/Upper Aptian
Little Sheep Mudstone Member (Cloverly Formation), Aptian to Aptian
PBDB

Ausktribosphenos
Wonthaggi formation, Flat rocks, early-middle Aptian.
(Rich et al. 1997; Benson et al. 2012)

Bishops
Wonthaggi formation, Flat rocks, Aptian.

(Rich et al. 2001a)

Brasilitherium
Caturrita Formation, Rosario del Sul Group. Norian
(Bonaparte et al. 2003; Langer and Ferigolo 2013)

Brasilodon
As Brasilitherium

Castorocauda
Daohugou locality, Tiaojishan formation
(Ji et al. 2006)

Cifelliodon
Yellow cat member, Cedar mountain formation.
Two different U/Pb ages: 124.2+-2.6 and 139.7+-2.2
(Huttenlocker et al. 2018)

Cimolestes
Hainin Formation, Danian
Tiupampan mammal zone, Santa Lucía Formation, Danian
Ravenscrag Formation, Puercan
Frenchman Formation, Puercan
Jbel Guersif Formation (Subatlas Group), Thanetian
Hell Creek Formation, Puercan
Bear Formation, Puercan
Frenchman Formation, Lancian
Ravenscrag Formation, Lancian
Hell Creek Formation, Lancian
Lance Formation, Lancian
Ferris Formation, Lancian
PBDB

Cimolodontans
Age of **Kryptobaatar** used.
Djadochta formation. Campanian
(Wible and Rougier 2000)

Daulestes
Bissekty Formation, Middle Turonian
PBDB

Deltatheridium
Djadokhta Formation, Campanian, Mongolia.
(Butler and Kielan-Jaworowska 1973; Godefroit et al. 2008)

Didelphodon
(Horseshoe Canyon Formation), Edmontonian
Scollard Formation, Lancian
Frenchman Formation
Lancian of Montana
Hell Creek Formation, Lancian
Lancian, South Dakota
Lancian of Wyoming
PBDB

Docofossor
Nanshimen site, Hebei, Tiaojishan formation
(Luo et al. 2015b)

Dryolestes
Morrison formation.
148-155 Mya
(Kowallis et al. 1998)

Eleutherodon
Forest Marble Formation (Great Oolite Group), Late/Upper Bathonian
(Kielan-Jaworowska et al. 2005)

Eomaia
Yixian formation. 129.7-122.1
(Ji et al. 2002; Chang et al. 2009)

Eoungulatum
Bissekty Formation, Middle Turonian
Aitym Formation, Late/Upper Turonian
PBDB

Fruitafossor
Morrison formation
(Luo and Wible 2005)

Gobiconodon
Cloverly formation. Aptian-Albian.
(Jenkins Jr and Schaff 1988)

Gypsonictos
Judithian – Lancian
(Kielan-Jaworowska et al. 2005)

Hadrocodium
Zhangjiawa member (dark red beds), Lower Lufeng formation, Sinemurian.
(Luo et al. 1994; Luo et al. 2001)

Hahnodon
Ksar Metlili Formation, Berriasian
PBDB

Haldanodon
Guimarota coal mine. Kimmeridgian.
(Martin and Nowotny 2000)

Haramiyavia
Tait Bjerg Beds of the Fleming Fjord formation. Norian-Rhaetian.
(Clemmensen et al. 2016)

Henkelotherium
Guimarota Coal mine. Kimmeridgian.
(Ruf et al. 2009)

Holoclemensia
Middle Member (Antlers Formation), Late/Upper Aptian
Middle Member (Antlers Formation), Albian
Paluxy Formation, Albian
PBDB

Jeholodens
Yixian formation.
(Qiang et al. 1999)

Juramaia
Tiaojishan formation.
(Luo et al. 2011; Gao and Shubin 2012)

Kennalestes
Djadokhta Formation, Campanian (*Kennalestes gobiensis*)
PBDB

Kielantherium
Höövör locality, Mongolia. Aptian-Albian.
(Lopatin and Averianov 2007)

Kokopellia
Mussentuchit, Cedar mountain formation. 97-98 Mya
(Cifelli and de Muizon 1997; Garrison et al. 2007)

Kuehneodon
Alcobaça Formation, Kimmeridgian
Sobral Formation, Late/Upper Kimmeridgian
Praia Azul Member (Lourinhã Formation), Late/Upper Kimmeridgian
PBDB

Maiopatagium
Daxishan, Tiaojishan formation
(Meng et al. 2017)

Maotherium
Lujiatun Locality, Yixian formation.
(Ji et al. 2009)

Massetognathus
Chañares formation
PBDB, (Marsicano et al. 2016)

Megaconus
Daohugou, Tiaojishan Formation
(Zhou et al. 2013)

Megazostrodon
Upper Elliot formation, Pokane, Lesotho. Hettangian-Sinemurian
(Gow 1986; Butler 2005)

Millsodon
Forest Marble Formation. Late Bathonian
PBDB

Montanalestes
Cloverly formation
(Cifelli 1999)

Morganucodon
Zhangjiawa member, Lower Lufeng formation, Sinemurian.
Glamorgan fissure fills, particularly St Brides island. Rhaetian-Sinemurian
(Luo et al. 1994; Kielan-Jaworowska et al. 2004)

Murtoilestes
Murtoi formation. Late Barremian-Middle Aptian
PBDB

Nanolestes

Guimarota coal mine and Qigu formation, China (Oxfordian).
(Martin et al. 2010)

Pachygenelus
Upper Elliot formation. Hettangian-Sinemurian
(Gow 2001)

Pediomys
age for *P. elegans*
Scollard formation. Lancian
Hell Creek formation. Lancian
Lance Formation. Lancian
Frenchman formation. Lancian
Ravenscrag formation. Lancian
PBDB

Peramus
Durlston Bay, Lulworth formation. Middle Berriasian
(Riboulleau et al. 2007; Davis 2012)

Plagiaulacidae
Durlston Bay, Lulworth formation, Middle Berriasian
(Kielan-Jaworowska et al. 2004)

Priacodon
Morrison formation.
(Rasmussen and Callison 1981)

Probainognathus
Chañares formation. 236-234 Mya
(Romer 1970; Marsicano et al. 2016)

Prokennalestes
Khoobur locality. Aptian-Albian
(Wible et al. 2001)

Protungulatum
Frenchman formation. Lancian
Ravenscrag formation. Lancian
Denver Formation. Puercan
Hell Creek Formation. Puercan (*P. donnae*)
Hell Creek Formation. Late Maastrichtian (*P. gorgun, coombsi*)
Ferris formation. Puercan
China Butte Member (Fort Union Formation). Puercan
PBDB

Pseudotribos
Daohugou locality, Tiaojishan formation
(Luo et al. 2007b)

Pucadelphys
Tiupamapa, Santa Lucia formation. 65MyA
(Macrini et al. 2007; Muizon et al. 2015)

Repenomamus
Yixian formation
(Li et al. 2001)

Rugosodon
Tiaojishan formation
(Yuan et al. 2013)

Shenshou
Daxishan, Tiaojoshan formation, Oxfordian
(Bi et al. 2014)

Shuotherium
Shangshaximiao formation. Oxfordian.
(Kielan-Jaworowska et al. 2004)

Sineleutherus
Qigu Formation, Oxfordian
Upper Member (Itat Formation), Bathonian
PBDB

Sinobaatar
Yixian formation. Fuxin formation. Aptian-Albian
(Hu and Wang 2002; Kusuhashi et al. 2009)

Sinoconodon
Zhangjiawa member (dark red beds), Lower Lufeng formation, Sinemurian
(Luo et al. 1994)

Sinodelphys
Yixian formation.
(Luo et al. 2003)

Spalacotherium
Purbeck limestone group and Wealden supergroup. Berriasian-Valanginian.
(Kielan-Jaworowska et al. 2004)

Spinolestes
La Huérguina Formation, Late/Upper Barremian
PBDB

Steropodon
Lightning ridge. Early-Mid Albian
(Archer et al. 1985; Kear and Godthelp 2008)

Sulestes
Bissekty formation. Middle–Upper Turonian
(Kielan-Jaworowska et al. 2004)

Teinolophos
Flat rocks, Victoria
(Rich et al. 2001b)

Thomasia
Early Rhaetian, Lorraine
? norian, Holwell, Britain
Late Rhaetian, Wurttemberg, Germany
(Kielan-Jaworowska et al. 2004)

Thrinaxodon
Lystrosaurus assemblage zone, Karoo. Induan-early Olenekian
(Estes 1961; Ezcurra et al. 2013)

Tinodon
Morrison formation and Purbeck limestone.
(Kielan-Jaworowska et al. 2004)

Trioracodon
Morrison formation and Purbeck limestone
(Kielan-Jaworowska et al. 2004)

Tritylodontidae
Age of Bienotherium and Oligokyphus used.
Bienotherium
Shawan member, Lower Lufeng formation, Hettangian.
(Luo et al. 1994)
Oligokyphus
Shawan member (Dull purplish) Lower Lufeng formation. Hettangian.
Windsor Hill fissure fill. Pleinsbachian
(Kühne 1956; Luo and Sun 1994)

Turgidodon
Oldman formation. Judithian
Dinosaur Park formation. Judithian
St. Mary River Formation, Edmontonian
Scollard Formation. Lancian
Foremost formation. Middle Campanian
Oldman Formation, Middle–Late Campanian
Frenchman Formation. Lancian
Judith River Formation. Middle Campanian
Hell Creek Formation. Lancian
Upper Shale Member (Aguja Formation), Judithian
Kaiparowits Formation, Late/Upper Campanian
Wahweap Formation, Judithian
Mesaverde Formation, Judithian
Lance Formation. Lancian
PBDB

Ukhaatherium
Djadochta formation. Campanian
(Horovitz 2003)

Vilevolodon
Nanshimen site, Tiaojishan formation
(Luo et al. 2017)

Vincelestes
La Amarga formation. Barremian
(Apesteguía 2007)

Vintana
La Kinkoky member, Maevarano formation. Maastrichian
(Krause et al. 2014)

Xianshou linglong
Daxishan, Tiaojoshan formation, Oxfordian
(Bi et al. 2014)

Xianshou songae
Daxishan, Tiaojoshan formation, Oxfordian
(Bi et al. 2014)

Yanoconodon
Yixian formation.
(Luo et al. 2007a)

Zhangheotherium
Yixian formation
(Hu et al. 1997)

Additional taxa for the Krause *et al.* matrix

Bharattherium
Intertrappean beds, Maastrichian.
(Prasad et al. 2007)

Bienotherium
Shawan member, Lower Lufeng formation, Hettangian.
(Luo et al. 1994)

Bienotheroides
Upper Xiashaximiao formation. Middle Jurassic.
(Sun and Li 1985; Danilov and Parham 2008)

Bocatherium
La Boca formation. Pleinsbachian.
(Montellano et al. 2008)

Catopsbaatar
Baruungoyot Formation equivalent. Campanian-Maastrichian.
(Hurum and Kielan-Jaworowska 2008)

Chulsanbaatar
Barun Goyot formation.
(Hurum 1998)

Cronopio
Candeleros Formation. Cenomanian.
(Rougier et al. 2011)

Cynognathus
Early to middle Triassic Burgersdorp formation (Karoo), and Mendoza. Cynognathus assemblage zone. Olenekian-Anisian.
(Abdala et al. 2005; Lucas 2010)

Diademodon
As Cynognathus
(Botha et al. 2005)

Gomphos

Nomogen formation and Arshanto formations, inner Mongolia. Palaeocene-Eocene boundary.
(Meng et al. 2004)

Gondwanatherium
Los Alamitos formation, Argentina. Campanian.
(Bonaparte 1986)

Greniodon
La Barda, Andesitas Huancache Formation, Argentina. Early Lutetian.
(Goin et al. 2012)

Guimarota Paulchoffatiidae
Guimarota coal mine. Kimmeridgian.
(Schwarz and Salisbury 2005)

Exaeretodon
Ischigualasto formation Argentina, and Santa Maria formation Brazil. Ladinian-Carnian.
(Abdala et al. 2002)

Ferugliotherium
Los Alamitos formation, Argentina. Campanian.
(Krause et al. 1992)

Henosferus
Canadon asfalto formation.
(Rougier et al. 2007)

Kayentatherium
Kayenta formation. Sinemurian-Pleinsbachian.
(Kermack 1982; Tykoski et al. 2002)

Kryptobaatar
Djadochta formation. Campanian
(Wible and Rougier 2000)

Lambdopsalis
Nomogen formation. Late Palaeocene
(Mao et al. 2015)

Lavanify
Maevarano Formation. Maastrichian
(Krause et al. 1997; Rogers et al. 2007)

Nemegtbaatar
Barun Goyot formation

(Hurum 1998)

Oligokyphus
Shawan member (Dull purplish) Lower Lufeng formation. Hettangian.
Windsor Hill fissure fill. Pleinsbachian
(Kühne 1956; Luo and Sun 1994)

Pappotherium
Butler farm local fauna, Albian
(Fox 1975)

Ptilodus
New Mexico, Torrejonian (Late Danian)
Fort Union, Montana, Torrejonian
Washakio basin, Wyoming, Torrejonian
Williston Basin Saskatchewan, Tiffanian (Selandian-Thanetian)
Cochrane, Alberta. Tiffanian
(Granger and Simpson 1929; Szalay 1965; Krause 1982; Scott et al. 2002)

Sudamerica
Punto Perigro, Salamanca formation. Danian
(Koenigswald et al. 1999; Woodburne et al. 2014)

Taeniolabis
Nacimento formation (Puercan)
Ludlow formation and Tullock formation, Montana. 65.5 Mya
(Simmons 1987; Greenwald 1988; Williamson et al. 2008)

Trapalcotherium
Allen formation. ? Maastrichian
(Rougier et al. 2009)

Tribosphenomys
Subeng, Inner Mongolia and Zhigden member, Naran Bulak formation, Mongolia. Thanetian
(Lopatin et al. 2004)

Tritheledontidae
Pachygenelus: Upper Elliot formation.
Brasilitherium and Brasilodon from the Caturrita Formation, Rosario del Sul Group. Norian
(Gow 2001; Bonaparte et al. 2003; Langer and Ferigolo 2013)

Yunnanodon
Zhangjiawa member (dark red beds), Lower Lufeng formation, Sinemurian.
(Fraser and Sues 1997)

Zalambdalestes
Djadochta formation.
(Wible et al. 2004)

Additional taxa for the Wang *et al.* matrix

Jeholbaatar
Jiufotang formation. 120.3+-0.7
(He et al. 2004; Wang et al. 2019)

Liaoconodon
Jiufotang formation.
(Meng et al. 2011)

Mayulestes
Tiupampan mammal zone, Santa Lucía Formation, Danian
PBDB

Supplementary References

Abdala, F., Barberena, M. C. and Dornelles, J. (2002). A new species of the traversodontid cynodont Exaeretodon from the Santa Maria Formation (Middle/Late Triassic) of southern Brazil. *Journal of Vertebrate Paleontology* **22,** 313–325.

Abdala, F., Hancox, P. J. and Neveling, J. (2005). Cynodonts from the uppermost Burgersdorp Formation, South Africa, and their bearing on the biostratigraphy and correlation of the Triassic Cynognathus Assemblage Zone. *Journal of Vertebrate Paleontology* **25,** 192–199.

Apesteguía, S. (2007). The sauropod diversity of the La Amarga Formation (Barremian), Neuquén (Argentina). *Gondwana Research* **12,** 533–546.

Archer, M., Flannery, T. F., Ritchie, A. and Molnar, R. (1985). First Mesozoic mammal from Australia—an early Cretaceous monotreme. *Nature* **318,** 363–366.

Benson, R. B., Rich, T. H., Vickers-Rich, P. and Hall, M. (2012). Theropod fauna from southern Australia indicates high polar diversity and climate-driven dinosaur provinciality. *PloS one* **7,** e37122.

Bi, S., Wang, Y., Guan, J., Sheng, X. and Meng, J. (2014). Three new Jurassic euharamiyidan species reinforce early divergence of mammals. *Nature* **514,** 579.

Bonaparte, J. F. (1986). A new and unusual Late Cretaceous mammal from Patagonia. *Journal of Vertebrate Paleontology* **6,** 264–270.

Bonaparte, J. F., Martinelli, A. G., Schultz, C. L. and Rubert, R. (2003). The sister group of mammals: small cynodonts from the Late Triassic of southern Brazil. *Revista Brasileira de Paleontologia* **5**.

Botha, J., Lee-Thorp, J. and Chinsamy, A. (2005). The palaeoecology of the non-mammalian cynodonts Diademodon and Cynognathus from the Karoo Basin of South Africa, using stable light isotope analysis. *Palaeogeography, Palaeoclimatology, Palaeoecology* **223,** 303–316.

Butler, P. and Clemens, W. (2001). Dental Morphology of the Jurassic Holotherian Mammal Amphitherium, with a Discussion of the Evolution of Mammalian Post-Canine Dental Formulae. *Palaeontology* **44,** 1–20.

Butler, P. and Kielan-Jaworowska, Z. (1973). Is Deltatheridium a marsupial?

Butler, P. M. and Sigogneau-Russell, D. (2016). Diversity of triconodonts in the Middle Jurassic of Great Britain. *PDF). Palaeontologia Polonica* **67,** 35–65.

Butler, R. J. (2005). The 'fabrosaurid'ornithischian dinosaurs of the upper Elliot Formation (Lower Jurassic) of South Africa and Lesotho. *Zoological Journal of the Linnean Society* **145,** 175–218.

Chang, S.-c., Zhang, H., Renne, P. R. and Fang, Y. (2009). High-precision 40 Ar/39 Ar age for the Jehol biota. *Palaeogeography, Palaeoclimatology, Palaeoecology* **280,** 94–104.

Cifelli, R. L. (1999). Tribosphenic mammal from the North American early Cretaceous. *Nature* **401,** 363–366.

Cifelli, R. L. and de Muizon, C. (1997). Dentition and jaw of Kokopellia juddi, a primitive marsupial or near-marsupial from the medial Cretaceous of Utah. *Journal of Mammalian Evolution* **4,** 241–258.

Clemmensen, L. B., Milàn, J., Adolfssen, J. S., Estrup, E. J., Frobøse, N., Klein, N., Mateus, O. and Wings, O. (2016). The vertebrate-bearing Late Triassic Fleming Fjord Formation of central East Greenland revisited: stratigraphy, palaeoclimate and new palaeontological data. *Geological Society, London, Special Publications* **434,** 31–47.

Cohen, K. M., Harper, D. A. T. and Gibbard, P. L. (2018). ICS International Chronostratigraphic Chart 2018/08. *International Commission on Stratigraphy, IUGS.* http://www.stratigraphy.org.

Cúneo, N. R. and Bowring, S. (2010). Dataciones geocronológicas preliminares en la Cuenca Cañadón Asfalto, Jurásico de Chubut, Argentina. In *X Congreso Argentino de Paleontología y Bioestratigrafía-VII Congreso Latinoamericano de Paleontología*.

Danilov, I. G. and Parham, J. F. (2008). A reassessment of some poorly known turtles from the Middle Jurassic of China, with comments on the antiquity of extant turtles. *Journal of Vertebrate Paleontology* **28,** 306–318.

Davis, B. M. (2012). Micro-computed tomography reveals a diversity of Peramuran mammals from the Purbeck Group (Berriasian) of England. *Palaeontology* **55,** 789–817.

Estes, R. (1961). *Cranial anatomy of the cynodont reptile Thrinaxodon liorhinus*. The Museum.

Ezcurra, M. D., Butler, R. J. and Gower, D. J. (2013). 'Proterosuchia': the origin and early history of Archosauriformes. *Geological Society, London, Special Publications* **379,** 9–33.

Flynn, J. J., Parrish, J. M., Rakotosamimanana, B., Simpson, W. F. and Wyss, A. R. (1999). A middle Jurassic mammal from Madagascar. *Nature* **401,** 57–60.

Fox, R. C. (1975). Molar structure and function in the Early Cretaceous mammal Pappotherium: evolutionary implications for Mesozoic Theria. *Canadian Journal of Earth Sciences* **12,** 412–442.

Fraser, N. C. and Sues, H.-D. (1997). *In the shadow of the dinosaurs: early Mesozoic tetrapods*. Cambridge University Press.

Gao, K.-Q. and Shubin, N. H. (2012). Late Jurassic salamandroid from western Liaoning, China. *Proceedings of the National Academy of Sciences* **109,** 5767–5772.

Garrison, J. R., Brinkman, D., Nichols, D. J., Layer, P., Burge, D. and Thayn, D. (2007). A multidisciplinary study of the Lower Cretaceous Cedar Mountain Formation, Mussentuchit Wash, Utah: a determination of the paleoenvironment and paleoecology of the Eolambia caroljonesa dinosaur quarry. *Cretaceous Research* **28,** 461–494.

Godefroit, P., Currie, P. J., Hong, L., Yong, S. C. and Zhi-Ming, D. (2008). A new species of Velociraptor (Dinosauria: Dromaeosauridae) from the Upper Cretaceous of northern China. *Journal of Vertebrate Paleontology* **28,** 432–438.

Goin, F. J., Tejedor, M. F., Chornogubsky, L., López, G. M., Gelfo, J. N., Bond, M., Woodburne, M. O., Gurovich, Y. and Reguero, M. (2012). Persistence of a Mesozoic, non-therian mammalian lineage (Gondwanatheria) in the mid-Paleogene of Patagonia. *Naturwissenschaften* **99,** 449–463.

Gow, C. E. (1986). A new skull of Megazostrodon (Mammalia, Triconodonta) from the Elliot Formation (Lower Jurassic) of southern Africa.

Gow, C. E. (2001). A partial skeleton of the tritheledontid Pachygenelus (Therapsida: Cynodontia).

Granger, W. and Simpson, G. G. (1929). *A revision of the Tertiary Multituberculata*. American Museum of Natural History.

Greenwald, N. S. (1988). Patterns of tooth eruption and replacement in multituberculate mammals. *Journal of Vertebrate Paleontology* **8,** 265–277.

Gurovich, Y. and Beck, R. M. D. (2009). The phylogenetic affinities of the enigmatic mammalian clade Gondwanatheria. *Journal of Mammalian Evolution* **16,** 25-49.

He, H. Y., Wang, X. L., Zhou, Z. H., Wang, F., Boven, A., Shi, G. H. and Zhu, R. X. (2004). Timing of the Jiufotang Formation (Jehol Group) in Liaoning, northeastern China, and its implications. *Geophysical Research Letters* **31,** 1709.

Horovitz, I. (2003). Postcranial skeleton of Ukhaatherium nessovi (Eutheria, Mammalia) from the late Cretaceous of Mongolia. *Journal of Vertebrate Paleontology* **23,** 857–868.

Hu, Y. and Wang, Y. (2002). Sinobaatar gen. nov.: first multituberculate from the Jehol Biota of Liaoning, northeast China. *Chinese Science Bulletin* **47,** 933–938.

Hu, Y., Wang, Y., Luo, Z. and Li, C. (1997). A new symmetrodont mammal from China and its implications for mammalian evolution. *Nature* **390,** 137–142.

Hurum, J. H. (1998). The inner ear of two Late Cretaceous multituberculate mammals, and its implications for multituberculate hearing. *Journal of Mammalian Evolution* **5,** 65–93.

Hurum, J. H. and Kielan-Jaworowska, Z. (2008). Postcranial skeleton of a Cretaceous multituberculate mammal Catopsbaatar. *Acta Palaeontologica Polonica* **53,** 545–566.

Huttenlocker, A. K., Grossnickle, D. M., Kirkland, J. I., Schultz, J. A. and Luo, Z.-X. (2018). Late-surviving stem mammal links the lowermost Cretaceous of North America and Gondwana. *Nature* **558,** 108.

Jenkins, F. A., Gatesy, S. M., Shubin, N. H. and Amaral, W. W. (1997). Haramiyids and Triassic mammalian evolution. *Nature* **385,** 715-718.

Jenkins Jr, F. A. and Schaff, C. R. (1988). The Early Cretaceous mammal Gobiconodon (Mammalia, Triconodonta) from the Cloverly Formation in Montana. *Journal of Vertebrate Paleontology* **8,** 1–24.

Ji, Q., Luo, Z.-X., Yuan, C.-X. and Tabrum, A. R. (2006). A swimming mammaliaform from the Middle Jurassic and ecomorphological diversification of early mammals. *Science* **311,** 1123–1127.

Ji, Q., Luo, Z.-X., Yuan, C.-X., Wible, J. R., Zhang, J.-P. and Georgi, J. A. (2002). The earliest known eutherian mammal. *Nature* **416,** 816–822.

Ji, Q., Luo, Z.-X., Zhang, X., Yuan, C.-X. and Xu, L. (2009). Evolutionary development of the middle ear in Mesozoic therian mammals. *Science* **326,** 278–281.

Kear, B. P. and Godthelp, H. (2008). Inferred vertebrate bite marks on an Early Cretaceous unionoid bivalve from Lightning Ridge, New South Wales, Australia. *Alcheringa* **32,** 65–71.

Kemp, T. S. (1982). *Mammal-like reptiles and the origin of mammals*. Academic Press, London.

Kemp, T. S. (2005). *The origin and evolution of mammals*. Oxford University Press, Oxford.

Kermack, D. (1982). A new tritylodontid from the Kayenta Formation of Arizona. *Zoological Journal of the Linnean Society* **76,** 1–17.

Kielan-Jaworowska, Z. (1975). Preliminary description of two new eutherian genera from the Late Cretaceous of Mongolia. *Palaeontologia Polonica* **33,** 5–16.

Kielan-Jaworowska, Z., Cifelli, R. L. and Luo, Z.-X. (2004). *Mammals from the age of dinosaurs: origins, evolution, and structure*. Columbia University Press.

Kielan-Jaworowska, Z., Cifelli, R. L. and Luo, Z.-X. (2005). *Mammals from the age of dinosaurs: origins, evolution, and structure*. Columbia University Press, New York.

Koenigswald, W. v., Goin, F. and Pascual, R. (1999). Hypsodonty and enamel microstructure in the Paleocene gondwanatherian mammal Sudamerica ameghinoi. *Acta Palaeontologica Polonica* **44,** 263–300.

Kowallis, B. J., Christiansen, E. H., Deino, A. L., Peterson, F., Turner, C. E., Kunk, M. J. and Obradovich, J. D. (1998). The age of the Morrison Formation. *Modern Geology* **22,** 235–260.

Krause, D. W. (1982). Jaw movement, dental function, and diet in the Paleocene multituberculate Ptilodus. *Paleobiology* **8,** 265–281.

Krause, D. W., Hoffmann, S., Wible, J. R., Kirk, E. C., Schultz, J. A., von Koenigswald, W., Groenke, J. R., Rossie, J. B., O'Connor, P. M. and Seiffert, E. R. (2014). First cranial remains of a gondwanatherian mammal reveal remarkable mosaicism. *Nature* **515,** 512–517.

Krause, D. W., Kielan-Jaworowska, Z. and Bonaparte, J. F. (1992). Ferugliotherium Bonaparte, the first known multituberculate from South America. *Journal of Vertebrate Paleontology* **12,** 351–376.

Krause, D. W., Prasad, G., von Koenigswald, W., Sahni, A. and Grine, F. E. (1997). Cosmopolitanism among Gondwanan late Cretaceous mammals. *Nature* **390,** 504–507.

Kühne, W. G. (1956). *The liassic therapsid Oligokyphus*. British Museum.

Kusuhashi, N., Hu, Y., Wang, Y., Setoguchi, T. and Matsuoka, H. (2009). Two Eobaatarid (Multituberculata; Mammalia) genera from the Lower Cretaceous Shahai and Fuxin Formations, northeastern Chinau. *Journal of Vertebrate Paleontology* **29,** 1264–1288.

Langer, M. C. and Ferigolo, J. (2013). The Late Triassic dinosauromorph Sacisaurus agudoensis (Caturrita Formation; Rio Grande do Sul, Brazil): anatomy and affinities. *Geological Society, London, Special Publications* **379,** 353–392.

Li, G. and Luo, Z.-X. (2006). A Cretaceous symmetrodont therian with some monotreme-like postcranial features. *Nature* **439,** 195–200.

Li, J., Wang, Y., Wang, Y. and Li, C. (2001). A new family of primitive mammal from the Mesozoic of western Liaoning, China. *Chinese Science Bulletin* **46,** 782–785.

Longrich, N. R., Currie, P. J. and ZHI-MING, D. (2010). A new oviraptorid (Dinosauria: Theropoda) from the Upper Cretaceous of Bayan Mandahu, Inner Mongolia. *Palaeontology* **53,** 945–960.

Lopatin, A. and Averianov, A. (2007). Kielantherium, a basal tribosphenic mammal from the Early Cretaceous of Mongolia, with new data on the aegialodontian dentition. *Acta Palaeontologica Polonica* **52**.

Lopatin, A. V., Averianov, A. O. and Lucas, S. (2004). A new species of Tribosphenomys (Mammalia: Rodentiaformes) from the Paleocene of Mongolia. *Paleogene mammals. New Mexico Museum of Natural History and Science Bulletin* **26,** 169–175.

Lucas, S. G. (2010). The Triassic timescale based on nonmarine tetrapod biostratigraphy and biochronology. *Geological Society, London, Special Publications* **334,** 447–500.

Lucas, S. G. and Luo, Z. (1993). Adelobasileus from the Upper Triassic of West Texas: the oldest mammal. *Journal of Vertebrate Paleontology* **13,** 309–334.

Luo, Z., Kielan-Jaworowska, Z. and Cifelli, R. (2004). Evolution of dental replacement in mammals. *Bulletin of Carnegie Museum of Natural History* **36,** 159-175.

Luo, Z. and Sun, A. (1994). Oligokyphus (Cynodontia: Tritylodontidae) from the Lower Lufeng Formation (Lower Jurassic) of Yunnan, China. *Journal of Vertebrate Paleontology* **13,** 477–482.

Luo, Z.-X., Chen, P., Li, G. and Chen, M. (2007a). A new eutriconodont mammal and evolutionary development in early mammals. *Nature* **446,** 288–293.

Luo, Z.-X., Crompton, A. W. and Sun, A.-L. (2001). A new mammaliaform from the early Jurassic and evolution of mammalian characteristics. *Science* **292,** 1535–1540.

Luo, Z.-X., Gatesy, S. M., Jenkins, F. A., Amaral, W. W. and Shubin, N. H. (2015a). Mandibular and dental characteristics of Late Triassic mammaliaform *Haramiyavia* and their ramifications for basal mammal evolution. *Proceedings of the National Academy of Sciences* **112,** E7101–E7109.

Luo, Z.-X., Ji, Q., Wible, J. R. and Yuan, C.-X. (2003). An Early Cretaceous tribosphenic mammal and metatherian evolution. *Science* **302,** 1934–1940.

Luo, Z.-X., Ji, Q. and Yuan, C.-X. (2007b). Convergent dental adaptations in pseudo-tribosphenic and tribosphenic mammals. *Nature* **450,** 93–97.

Luo, Z.-X., Meng, Q.-J., Grossnickle, D. M., Liu, D., Neander, A. I., Zhang, Y.-G. and Ji, Q. (2017). New evidence for mammaliaform ear evolution and feeding adaptation in a Jurassic ecosystem. *Nature* **548,** 326.

Luo, Z.-X., Meng, Q.-J., Ji, Q., Liu, D., Zhang, Y.-G. and Neander, A. I. (2015b). Evolutionary development in basal mammaliaforms as revealed by a docodontan. *Science* **347,** 760-764.

Luo, Z.-X. and Wible, J. R. (2005). A Late Jurassic digging mammal and early mammalian diversification. *Science* **308,** 103–107.

Luo, Z.-x., Wu, X.-C., Fraser, N. and Sues, H. (1994). The small tetrapods of the lower Lufeng Formation, Yunnan, China. *The Shadow of the Dinosaurs: Early Mesozoic Tetrapods***,** 251–270.

Luo, Z.-X., Yuan, C.-X., Meng, Q.-J. and Ji, Q. (2011). A Jurassic eutherian mammal and divergence of marsupials and placentals. *Nature* **476,** 442–445.

Macrini, T. E., De Muizon, C., Cifelli, R. L. and Rowe, T. (2007). Digital cranial endocast of Pucadelphys andinus, a Paleocene metatherian. *Journal of Vertebrate Paleontology* **27,** 99–107.

Mao, F., Wang, Y. and Meng, J. (2015). A Systematic Study on Tooth Enamel Microstructures of Lambdopsalis bulla (Multituberculate, Mammalia)-Implications for Multituberculate Biology and Phylogeny. *PloS one* **10,** e0128243.

Marsicano, C. A., Irmis, R. B., Mancuso, A. C., Mundil, R. and Chemale, F. (2016). The precise temporal calibration of dinosaur origins. *Proceedings of the National Academy of Sciences* **113,** 509–513.

Martin, T., Averianov, A. O. and Pfretzschner, H.-U. (2010). Mammals from the Late Jurassic Qigu Formation in the southern Junggar Basin, Xinjiang, Northwest China. *Palaeobiodiversity and Palaeoenvironments* **90,** 295–319.

Martin, T. and Nowotny, M. (2000). The docodont Haldanodon from the Guimarota mine. *Guimarota—a Ju– rassic Ecosystems*, 91–96.
Meng, J., Bowen, G. J., Jie, Y., Koch, P. L., Ting, S., Qian, L. and Jin, X. (2004). *Gomphos elkema* (Glires, Mammalia) from the Erlian Basin: Evidence for the Early Tertiary Bumbanian Land Mammal Age in Nei-Mongol, China. *American Museum Novitates*, 1–24.
Meng, J., Wang, Y. and Li, C. (2011). Transitional mammalian middle ear from a new Cretaceous Jehol eutriconodont. *Nature* **472,** 181-185.
Meng, Q.-J., Grossnickle, D. M., Liu, D., Zhang, Y.-G., Neander, A. I., Ji, Q. and Luo, Z.-X. (2017). New gliding mammaliaforms from the Jurassic. *Nature* **548,** 291-296.
Meng, Q.-J., Ji, Q., Zhang, Y.-G., Liu, D., Grossnickle, D. M. and Luo, Z.-X. (2015). An arboreal docodont from the Jurassic and mammaliaform ecological diversification. *Science* **347,** 764-768.
Montellano, M., Hopson, J. A. and Clark, J. M. (2008). Late Early Jurassic mammaliaforms from Huizachal Canyon, Tamaulipas, México. *Journal of Vertebrate Paleontology* **28,** 1130–1143.
Muizon, C. D., Billet, G., Argot, C., Ladevèze, S. and Goussard, F. (2015). Alcidedorbignya inopinata, a basal pantodont (Placentalia, Mammalia) from the early Palaeocene of Bolivia: anatomy, phylogeny and palaeobiology. *Geodiversitas* **37,** 397–634.
Paradis, E., Claude, J. and Strimmer, K. (2004). APE: analyses of phylogenetics and evolution in R language. *Bioinformatics* **20,** 289–290.
Parker, W. G., Stocker, M. R. and Irmis, R. B. (2008). A new desmatosuchine aetosaur (Archosauria: Suchia) from the Upper Triassic Tecovas Formation (Dockum Group) of Texas. *Journal of Vertebrate Paleontology* **28,** 692–701.
Prasad, G. V., Verma, O., Sahni, A., Krause, D. W., Khosla, A. and Parmar, V. (2007). A new Late Cretaceous gondwanatherian mammal from central India. *Proceedings-indian national science academy* **73,** 17.
Qiang, J., Zhexi, L. and Shu-An, J. (1999). A Chinese triconodont mammal and mosaic evolution of the mammalian skeleton. *Nature* **398,** 326–330.
Rasmussen, T. and Callison, G. (1981). A new species of triconodont mammal from the Upper Jurassic of Colorado. *Journal of Paleontology*, 628–634.
Rauhut, O. W., Martin, T., Ortiz-Jaureguizar, E. and Puerta, P. (2002). A Jurassic mammal from South America. *Nature* **416,** 165–168.
Riboulleau, A., Schnyder, J., Riquier, L., Lefebvre, V., Baudin, F. and Deconinck, J.-F. (2007). Environmental change during the Early Cretaceous in the Purbeck-type Durlston Bay section (Dorset, Southern England): a biomarker approach. *Organic Geochemistry* **38,** 1804–1823.
Rich, T., Flannery, T., Trusler, P., Kool, L., Van Klaurer, N. and Vickers-Rich, P. (2001a). A second tribosphenic mammal from the Mesozoic of Australia. *Records of the Queen Victoria Museum* **110,** 1–9.
Rich, T. H., Vickers-Rich, P., Constantine, A., Flannery, T. F., Kool, L. and van Klaveren, N. (1997). A tribosphenic mammal from the Mesozoic of Australia. *Science* **278,** 1438–1442.
Rich, T. H., Vickers-Rich, P., Trusler, P., Flannery, T. F., Cifelli, R., Constantine, A., Kool, L. and Van Klaveren, N. (2001b). Monotreme nature of the Australian Early Cretaceous mammal Teinolophos. *Acta Palaeontologica Polonica* **46**.
Rogers, R. R., Krause, D. W., Rogers, K. C., Rasoamiaramanana, A. H. and Rahantarisoa, L. (2007). Paleoenvironment and paleoecology of Majungasaurus crenatissimus (Theropoda: Abelisauridae) from the Late Cretaceous of Madagascar. *Journal of Vertebrate Paleontology* **27,** 21–31.
Romer, A. S. (1970). *The Chãnares (Argentina) Triassic Reptile Fauna: A Chiniquodontid Cynodont with an Incipient Squamosal-dentary Jaw Articulation. VI*. Museum of Comparative Zoology.
Rougier, G. W., Apesteguía, S. and Gaetano, L. C. (2011). Highly specialized mammalian skulls from the Late Cretaceous of South America. *Nature* **479,** 98–102.
Rougier, G. W., Chornogubsky, L., Casadio, S., Arango, N. P. and Giallombardo, A. (2009). Mammals from the Allen Formation, Late Cretaceous, Argentina. *Cretaceous Research* **30,** 223–238.

Rougier, G. W., Martinelli, A. n. G., Forasiepi, A. a. M. and Novacek, M. J. (2007). New Jurassic mammals from Patagonia, Argentina: a reappraisal of australosphenidan morphology and interrelationships. *American Museum Novitates* **3566,** 1-54.

Ruf, I., Luo, Z. X., Wible, J. R. and Martin, T. (2009). Petrosal anatomy and inner ear structures of the Late Jurassic Henkelotherium (Mammalia, Cladotheria, Dryolestoidea): insight into the early evolution of the ear region in cladotherian mammals. *Journal of Anatomy* **214,** 679–693.

Schliep, K. P. (2010). phangorn: phylogenetic analysis in R. *Bioinformatics***,** btq706.

Schwarz, D. and Salisbury, S. W. (2005). A new species of Theriosuchus (Atoposauridae, Crocodylomorpha) from the Late Jurassic (Kimmeridgian) of Guimarota, Portugal. *Geobios* **38,** 779–802.

Scott, C. S., Fox, R. C. and Youzwyshyn, G. P. (2002). New earliest Tiffanian [Late Paleocene] mammals from Cochrane 2, Southwestern Alberta, Canada. *Acta Palaeontologica Polonica* **47**.

Simmons, N. B. (1987). A revision of Taeniolabis (Mammalia: Multituberculata), with a new species from the Puercan of eastern Montana. *Journal of Paleontology* **61,** 794–808.

Sun, A. and Li, Y. (1985). The postcranial skeleton of the late tritylodont Bienotheroides. *Vertebrata PalAsiatica* **23**.

Szalay, F. S. (1965). First evidence of tooth replacement in the subclass Allotheria (Mammalia). American Museum novitates; no. 2226.

Szalay, F. S. and Trofimov, B. A. (1996). The Mongolian Late Cretaceous Asiatherium, and the early phylogeny and paleobiogeography of Metatheria. *Journal of Vertebrate Paleontology* **16,** 474–509.

Turner, A. H., Pritchard, A. C. and Matzke, N. J. (2017). Empirical and Bayesian approaches to fossil-only divergence times: A study across three reptile clades. *PloS one* **12,** e0169885.

Tykoski, R. S., Rowe, T. B., Ketcham, R. A. and Colbert, M. W. (2002). Calsoyasuchus valliceps, a new crocodyliform from the Early Jurassic Kayenta Formation of Arizona. *Journal of Vertebrate Paleontology* **22,** 593–611.

Velazco, P. M., Buczek, A. J. and Novacek, M. J. (2017). Two new tritylodontids (Synapsida, Cynodontia, Mammaliamorpha) from the Upper Jurassic, Southwestern Mongolia. *American Museum Novitates***,** 1-35.

Wang, H., Meng, J. and Wang, Y. (2019). Cretaceous fossil reveals a new pattern in mammalian middle ear evolution. *Nature* **576,** 102-105.

Wible, J. R., Novacek, M. J. and Rougier, G. W. (2004). New data on the skull and dentition in the Mongolian Late Cretaceous eutherian mammal Zalambdalestes. *Bulletin of the American Museum of Natural History***,** 1–144.

Wible, J. R. and Rougier, G. W. (2000). Cranial anatomy of Kryptobaatar dashzevegi (Mammalia, Multituberculata), and its bearing on the evolution of mammalian characters. *Bulletin of the American Museum of Natural History***,** 1–120.

Wible, J. R., Rougier, G. W., Novacek, M. J. and McKENNA, M. C. (2001). Earliest eutherian ear region: a petrosal referred to Prokennalestes from the Early Cretaceous of Mongolia. *American Museum Novitates***,** 1–44.

Williamson, T. E., Nichols, D. J. and Weil, A. (2008). Paleocene palynomorph assemblages from the Nacimiento Formation, San Juan Basin, New Mexico, and their biostratigraphic significance. *New Mexico Geology* **30**.

Woodburne, M. O., Goin, F. J., Raigemborn, M. S., Heizler, M., Gelfo, J. N. and Oliveira, E. V. (2014). Revised timing of the South American early Paleogene land mammal ages. *Journal of South American Earth Sciences* **54,** 109–119.

Yu, G., Smith, D. K., Zhu, H., Guan, Y. and Lam, T. T. Y. (2017). ggtree: an R package for visualization and annotation of phylogenetic trees with their covariates and other associated data. *Methods in Ecology and Evolution* **8,** 28-36.

Yuan, C.-X., Ji, Q., Meng, Q.-J., Tabrum, A. R. and Luo, Z.-X. (2013). Earliest evolution of multituberculate mammals revealed by a new Jurassic fossil. *Science* **341,** 779–783.

Zhang, C., Stadler, T., Klopfstein, S., Heath, T. A. and Ronquist, F. (2016). Total-evidence dating under the fossilized birth–death process. *Systematic Biology* **65,** 228–249.

Zhou, C.-F., Wu, S., Martin, T. and Luo, Z.-X. (2013). A Jurassic mammaliaform and the earliest mammalian evolutionary adaptations. *Nature* **500,** 163-167.

Zhou, Z.-H. and Wang, Y. (2017). Vertebrate assemblages of the Jurassic Yanliao Biota and the Early Cretaceous Jehol Biota: comparisons and implications. *Palaeoworld* **26,** 241-252.
